# Usability and Effectiveness of eHealth and mHealth Interventions That Support Self-Management and Health Care Transition in Adolescents and Young Adults With Chronic Disease: Systematic Review

**DOI:** 10.2196/56556

**Published:** 2024-11-26

**Authors:** ZhiRu Li, FangYan Lu, JingYun Wu, RuiJie Bao, YuXin Rao, Yun Yang, Huafen Wang

**Affiliations:** 1 Nursing Department The First Affiliated Hospital Zhejiang University School of Medicine Hangzhou China

**Keywords:** eHealth, mHealth, mobile health, chronic disease, healthcare transition, self-management, adolescents, young adults, chronic illness, systematic review, digital health, health education, social support, symptom tracking, monitoring, effectiveness

## Abstract

**Background:**

With advances in medical technologies, more children with chronic diseases are now living on into adulthood. The development of proficient self-management skills is essential for adolescents and young adults to transition from pediatric to adult health care services. An innovative way to improve the current care model and foster self-management skills could be through eHealth or mHealth (mobile health) interventions, in particular, when considering the rising ownership of digital technology by adolescents and young adults.

**Objective:**

This systematic review aimed to evaluate the features, acceptability, usability, engagement, and intervention efficacy of eHealth and mHealth interventions that support self-management and health care transition in adolescents and young adults with chronic disease.

**Methods:**

This review followed the PRISMA (Preferred Reporting Items for Systematic Reviews and Meta-Analyses) reporting guidelines (registration number CRD42023378355). We systematically searched the MEDLINE complete, Embase, Cochrane Library, CINAHL complete, and ProQuest Health & Medical complete. We considered only articles published in or since 2019, as we aimed to extend the data collected by 2 previous systematic reviews.

**Results:**

A total of 16,752 studies were screened. After removing duplicates, 14,507 studies were excluded based on the title and abstract. Ultimately, 22 studies were included. The interventions ranged from simple text messages to complex interventions involving web-based games and engagement of health care providers, which were summarized into 6 themes: medication monitoring and reminders, symptom tracking and monitoring, management goal setting, knowledge education and self-management skills training, incentives and reinforcement, and communication. Most adolescents and young adults felt the eHealth and mHealth interventions were feasible, as they were convenient, easy to use, and accessible in the context of helping manage their health. However, user engagement was variable and presented a gradual decline in youth engagement with these apps over time. Barriers that prevent user engagement are diverse, such as time-consuming video uploads, noncontinuous access to a phone, reading literacy levels, language, and false impressions. Moreover, adolescents and young adults had different preferred styles of message delivery and functions, especially the engaging elements, disease-specific information, and opportunities to communicate with peers, health care providers, and app teams.

**Conclusions:**

There remains limited data about the effectiveness of eHealth and mHealth interventions facilitating the self-management and health care transition of adolescents and young adults with chronic diseases. Based on the available evidence, they were receptive to and interested in receiving information and managing their health using mobile apps or websites. Considering adolescents and young adults had different preferred styles of message delivery and features, to improve user engagement and provide focused interventions, it would be better to involve them early in the design process to identify their specific needs, as well as collaborate with health care providers and app teams to obtain suggestions.

## Introduction

### Background

Chronic diseases in childhood refer to children aged 0-18 years who endure incurable diseases diagnosed by reproducible and valid methods according to professional standards, with a duration of longer than three months or a frequency of more than three times during the past year and will probably reoccur [1]. In recent years, the incidence of chronic diseases in children has significantly increased, which has become a major global public health issue affecting children’s physical and mental health [2,3]. Research showed that approximately 10% to 20% of children currently endure chronic diseases, including asthma, epilepsy, diabetes, chronic kidney disease, etc [4]. Due to development of the advanced medical technologies that support disease management and longer life spans, more children with chronic diseases are now living into adolescence and young adulthood [5]. To receive age-appropriate care, adolescents and young adults need to transition from pediatric to adult health care, which involves a significant shift in the care model from chronic disease supervision and management to self-management [6,7]. Pediatric providers tend to prefer a family-centered model of care, with parental involvement in disease management and daily care for children; while adult care, is patient-centered, emphasizing patient independence and personalized care [5,8]. Therefore, the health care transition from child-centered to adult-oriented health care systems is a period of increased risk and vulnerability.

Transition readiness, as a key element of health care transition, refers to the abilities to prepare, enter, continue, and complete the transition. Such preparation mainly reflects self-management ability, including the knowledge, skills, and experience required for disease management [9]. Self-management is an important skill for adolescent chronic disease patients to take over responsibility for their own health care and ensure good disease control [10,11]. Research has shown that inadequate self-management can lead to reduced adherence to treatment, decreased follow-up, and increased risk of mortality among adolescents and young adults with chronic diseases, thus placing a heavy economic burden on adolescents and young adults, their families, and even society [12-14]. Therefore, it is crucial to encourage and promote the improvement of adolescents’ and young adults’ self-management behaviors to ensure the continuity of their health management. However, changes in self-management behavior entail dynamic, continuous, and complex processes that are influenced by multiple factors, and these changes are often difficult to maintain [15]. Therefore, it is very important to find suitable methods to use in adolescent development to encourage and promote the improvement of self-management behavior among young people.

An innovative way to improve the current care model and foster self-management skills could take the form of digital health interventions, especially in light of the changing landscape of internet use and increasing ownership rates of digital technology among young people. Digital health intervention refers to the use of the internet, smartphones, social media, and mobile apps to provide health care information support and treatment to a target population [16]. Such interventions have come to represent an emerging mode of chronic disease health management due to the advantages of convenience, interactivity, accessibility, and low cost, which can effectively provide personalized and continuous web-based medical assurance [17]. Digital media have come to represent an indispensable aspect of young people’s lives, as young people rely on digital media to seek different forms of mental health support, relaxation, and distraction [18-20]. The use of eHealth or mHealth (mobile health) interventions to promote self-disease management among adolescents and young adults is an emerging field that is worthy of exploration, and this approach represents an innovative way to develop adolescents and young adults’ self-management skills and prepare them for the health care transition process [21,22].

To date, there has been little evidence produced about the effectiveness and usability of eHealth and mHealth interventions aimed at supporting self-management and health care transition in adolescents and young adults with chronic diseases. A systematic review conducted by Pérez et al [23] examined the literature published from 2015 to 2018 with the goal of evaluating the utility and effectiveness of mobile and web-based health apps that support self-management and transition among young people with chronic diseases, and 6 studies were ultimately included in that review. However, only a limited number of studies on this topic have been published, and it is difficult to draw comprehensive conclusions concerning the effectiveness and usage of mobile and web-based apps. Low and Manias [24] examined the usability of technology-based tools for supporting adolescents and young adults with chronic diseases by searching for data published between 1967 and 2019 in 3 electronic databases. However, among the included studies, most were published before 2015. Due to the rapid development of mobile medical technology and the increasingly mature forms of chronic disease management developed for adolescents and young adults, new interventions that are more suitable for self-management skill development may have emerged. In addition, although a meta-analysis was conducted in this study, the quantitative data was insufficient as it focused on the experiences and perspectives of adolescents and young adults [24].

### Objective

In summary, the effectiveness and usability of eHealth and mHealth interventions among adolescents and young adults with chronic diseases remain unclear. The aims of this systematic review are to provide more up-to-date evidence for the features and effectiveness of eHealth and mHealth interventions that support self-management and health care transition in adolescents and young adults with chronic diseases. In addition, to understand and optimize the users’ experience, we attempted to evaluate the acceptability, perceived level of usefulness, and user engagement of mHealth and eHealth interventions.

## Methods

### Study Design

The protocol for this systematic review was registered on PROSPERO (International Prospective Register of Systematic Review; registration number CRD42023378355). This study was reported per the PRISMA (Preferred Reporting Items for Systematic Reviews and Meta-Analyses) reporting guidelines [25].

### Search Strategy

We searched five electronic databases, namely MEDLINE complete, Embase, Cochrane Library, CINAHL complete, Web of Science, and ProQuest Health & Medical complete. The end date for article searching was January 2024. We identified studies published in or since 2019 in these databases. In addition to the mentioned search strategies, we also manually searched reference lists of included studies to identify any additional studies that fit the inclusion criteria. The search strategy (Multimedia Appendix 1) was developed in consultation with an information scientist and used standardized indexed search terms and free-text terms about the following four key concepts: (1) adolescents or young adults, (2) chronic conditions, (3) transition or disease management, and (4) technology.

### Inclusion and Exclusion Criteria

The studies included in this review met the inclusion criteria outlined in Textbox 1. The year of publication was limited between 2019 and 2024, as this review aims to update the 2 previous systematic reviews cited in the introductory section. The language restriction was attributed to the reviewers’ proficiency in English. Exclusion criteria included (1) eHealth and mHealth interventions about the management of mental health, pain, acute cancer, or health risk behaviors; (2) interventions used only for testing equipment, such as a Bluetooth spirometer and blood glucose monitors, or interventions focused on remote health monitoring, such as patient portals and symptom reporting platforms; and (3) less than 50% of the sample involved adolescents or young adults aged 10 to 24 years.

**Eligibility criteria.**

**Population**: Adolescents and young adults aged 10-24 years [26] who had been diagnosed with chronic conditions and who were either transitioning or had already transitioned to adult health care services.

**Interventions**: Any eHealth or mobile health interventions, such as digital tools, devices, systems, and resources, delivered through a web-based device to support the health care transition and aid adolescents and young adults in self-management.

**Comparisons**: Intervention versus ordinary care, pre-post or no comparator.

**Outcomes:** Any physiological, psychological, behavioral, attitudinal, or knowledge outcomes.

**Study Design**: Original quantitative or qualitative studies or mixed methods studies published in peer-reviewed journals in English.

**Year of publication**: Studies published in or since 2019.

### Study Selection

A PRISMA diagram illustrating the detailed study selection process is shown in the Results section (PRISMA checklist is provided in Multimedia Appendix 2). The search results were collated using Endnote X9 (Clarivate) software and filtered to eliminate duplicates. One reviewer (ZL) was responsible for the first stage of screening, which focused on titles and abstracts in light of the research questions and the inclusion and exclusion criteria. Then, 2 reviewers (JW and YR) screened abstracts for possible inclusion in the full-text screening. In all cases, the decision to include or exclude a single study was approved by both reviewers. If these 2 reviewers could not agree on the decision, a final decision was made by the third reviewer (FL).

### Data Extraction

A standardized table was used to extract the following data from each study: author information (name of the first author, year, and country), participant information (age, gender, and chronic condition), study characteristics (study design, sample size, duration, intervention media, intervention components, and quality assessment), and study outcomes. If any discrepancies between the reviewers emerged, they were resolved through discussion with the wider research team.

### Quality Assessment

The Downs and Black checklist for randomized and nonrandomized studies was used to appraise the quality of the intervention efficacy trials [27]. This checklist takes into account 5 main assessment areas, namely, reporting, external validity, internal validity based on bias, internal validity based on cofounding and selection bias, and power, and it assigns trials an overall score, with the highest possible score being 28. A score of 24 to 28 was graded excellent, 19 to 23 was graded good, 14 to 18 was graded fair, and <14 was graded poor. Additionally, we assessed the explicitness of reporting of each qualitative or quantitative questionnaire study to provide contextual details for readers to assess the transferability of this study’s findings to their own settings. The 21-item standards for reporting qualitative research (SRQR) checklist was used for qualitative research [28], with a total score of 0-21 (yes=1, partially=0.5, and no=0), and a higher score indicates higher quality. A 16-item tool developed by Tong et al [29] was used for questionnaire research, with the highest possible score being 16, and a higher score indicates higher quality. Two reviewers (RB and YY) assessed the quality of the included studies independently. Any disagreements between these two authors were resolved through discussion among the research group and re-examination of the studies.

### Data Synthesis and Analysis

Due to the heterogeneity exhibited by different studies and the different stages of intervention development, meta-analysis was deemed unsuitable for this systematic review. Instead, we used a narrative synthesis methodology to organize, explore, and present potential similarities and differences, associations, and patterns of data across different studies. For qualitative research, including exploratory and feasibility studies, mixed methods studies, and studies that involved interviews on focus groups, NVivo software (version 11; QSR International Pty Ltd) was used to extract and organize the data. The 3-stage thematic synthesis outlined by Thomas and Harden [30] was applied to the data synthesis and thematic analysis by one reviewer.

## Results

### Overview

**Figure 1:**
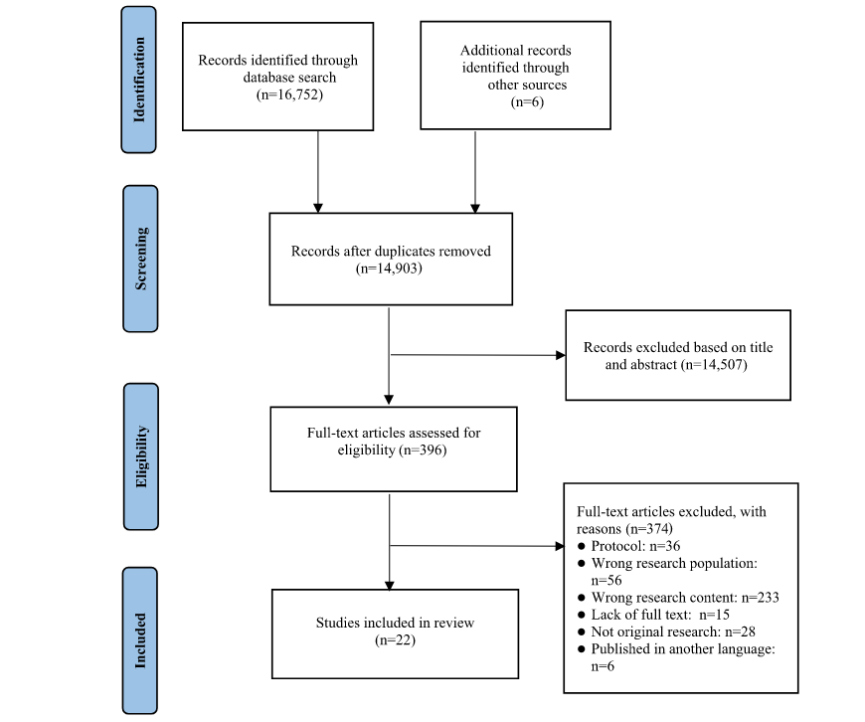
Study flow diagram.

Among a total of 16,758 studies identified based on the aforementioned search strategy, 1855 were removed due to duplication. Titles and abstracts of the remaining 14,903 articles were screened based on the inclusion criteria. Consequently, 14,507 articles were excluded, leaving 396 full-text papers that were reviewed for eligibility. Among them, 374 studies were excluded due to wrong research content (n=233), wrong research population (n=56), ineligible study designs (n=28), lack of full-text (n=15), protocol (n=36), and published in another language (n=6). Ultimately, 22 studies were included in the review [31-52] (Figure 1).

### Participants

This review included 1272 participants. Asthma was the most frequently investigated type of chronic disease (n=6) [31,35,36,39,43,51], followed by organ transplantation (n=4) [40,42,44,47], sickle cell disease (n=3) [33,34,37], type 1 diabetes (n=3) [38,41,50], inflammatory bowel disease (n=2) [45,48], cancer (n=1) [32], heart disease (n=1) [52], and HIV (n=1) [49]; 1 study included children with diabetes, those with asthma, those with cerebral palsy, and those with congenital cardiac conditions [46]. Study sample sizes ranged from 4 to 234 participants, who varied in terms of age (range 9-25 years) and gender (female: 562/1214, 46.3%). The characteristics of the included studies are shown in Table 1.

**Table 1:** Characteristics of the included studies (N=22).

Author (year)	Country	Chronic condition	Study design	Age (years)			Number of participants	Outcome measures	Quality assessment (scoring rate, %)
				Range	Mean	n (%)			
Kosse et al (2019) [31]	Netherlands	Asthma	RCT^a^	12-18	15.1	Male: 111(47.4)	234	Medication adherence^b^ Disease control^c^ Quality of life^d^	D&B^e^: 71.4
Schwartz et al (2019) [32]	United States	Survivors of Childhood Cancer	Multiphase iterative design and development of an intervention	Phase 2: 15-21 Phase 3: 15-29	Phase 2: 19 Phase 3: 18	Phase 2: male: 4(40) Phase 3: male: 1(12)	Phase 2: 10 Phase 3: 8	Usability and acceptability	Phase 2: D&B: 50 Phase 3: SRQR^f^: 57.1
Crosby et al (2020) [33]	United Kingdom	Sickle cell disease	RCT	13-21	16	Male: 25(47.1)	53	Self-efficacy^g^ Self-management skills^h^ SCD knowledge^i^ Health motivation^j^ Quality of life^k^	D&B: 75
Saulsberry et al (2020) [34]	United States	Sickle cell disease	A retrospective cohort study	12-25	14	Male: 113(62)	183	Disease knowledge^l^ Self-management confidence^m^	Tong: 42.9
Nichols et al (2020) [35]	United States	Asthma	A qualitative study	—^n^	Phase 1: 10.9 Phase 2: 12	Phase 1: male: 5(36) Phase 2: male: 6(75)	Phase 1: 14 Phase 2: 8	Challenges and benefits associated with SAMS Feasibility, acceptability, and preferences	SRQR: 83.3
Schneider et al (2020) [36]	United States	Asthma	A qualitative study	12-17	14.4	Male: 9(45)	20	Usability	SRQR: 78.6
Hood et al (2021) [37]	United Kingdom	Sickle cell disease	RCT	13-21	16.7	Male: 24(46)	52	User engagement^o^ Self-management skills^g^ Self-efficacy skills^p^	D&B: 53.6
Holtz et al (2021) [38]	United States	Type 1 diabetes	Pilot study (a preclinical or postclinical trial)	10-15	12.3	Male: 13(52)	25 adolescents and 25 parents	Adherence^q^ Quality of life^r^ Family conflict^s^ Satisfaction^t^ Use^u^ HbA1c levels^v^	D&B: 60.7
Davis et al (2021) [39]	Australia	Asthma	A pilot study	15-24	19.7	Male: 7(58)	12	Asthma control^w^ Asthma quality of life^x^ Usefulness, satisfaction, and acceptability^y^ Self-management^z^	SRQR: 45.2 Tong: 50
Brookshire-Gay et al (2021) [40]	United States	Hematopoietic cell transplantation	Quantitative feasibility pilot study using questionnaires	11-24	18.1	Male: 20(67)	30	Use^aa^ Global distress^ab^ Hospital readmission rates	Tong: 42.9
Butalia et al (2021) [41]	Canada	Type 1 diabetes	Nonrandomized trial	17-18	18	Male: 102(50.2)	203	Routine clinic visits HbA_1c_ Albumin and creatinine level ratio Emergency department visits and hospitalization rates	D&B: 71.4
Mehta et al (2021) [42]	United States	Liver transplant recipients	A qualitative study	12-18	—	Male: 3(75)	4	—	SRQR: 80.9
Fedele et al (2021) [43]	United States	Asthma	A pilot RCT	12-15	13.2	Male: 18(54.5)	33	Feasibility and acceptability Asthma management^ac^ Asthma control^ad^ Quality of life^d^ Self-efficacy^ae^ Family communication^af^	D&B: 60.7
Sayegh et al (2022) [44]	United States	Liver transplants	A pilot RCT	13-21	16	Male: 16(45.7)	35	Medication adherence^ag^ Adherence motivation^ah^ Immunosuppressant level^ai^ Feasibility and acceptability	D&B: 82.1
Daraiseh et al (2022) [45]	United States	Inflammatory bowel disease	Multiphase, participatory user research study using individual interviews, and user evaluation	Design phase: 14-25 Usability testing: 14-20	—	Male: 9(27.3)	Design phase: 14 Usability testing: 11	Usability^aj^ System usability scale^ak^	SRQR: 59.5 Tong: 57.1
Miller (2022) [46]	United States	Diabetes, asthma, cerebral palsy, and congenital cardiac disease	A pilot study	12-21	—	—	23	Knowledge of medical conditions, health care system navigation, and identified health care provider and section	SRQR: 52.4 Tong: 28.6
Kindem et al (2023) [47]	Norway	Solid organ transplantation	A preclinical or postclinical trial	14-25	14.5	Male: 8(40)	20	Feasibility Medication adherenceal Graft function	D&B: 67.9
Hommel et al (2023) [48]	United States	Inflammatory bowel disease	Multiphase, iterative design, and development of an intervention	9-18	14.1	Male: 13(62)	22	Adherence^am^ Feasibility and acceptability Quality of life^an^ Patient-reported symptoms^ao^	D&B: 42.9
Fomo et al (2023) [49]	United States	HIV	A qualitative study	14-19	16.2	Male: 9(60)	15	Perceived usefulness Facilitating conditions	SRQR: 80.9
Chiang et al (2023) [50]	China	Type 1 diabetes	Multiphase mixed methods design	16-25	—	—	35	User engagement satisfaction^ap^ Heuristic evaluation^aq^ Think-aloud evaluation^ar^	SRQR: 50 Tong: 64.3
Ghozali et al (2023) [51]	Indonesia	Asthma	A preclinical or postclinical trial	18-22	—	Male: 65(46.4)	140	Asthma control^as^	D&B: 53.6
Han et al (2023) [52]	Canada	Heart disease	RCT	16-18	—	Male: 40(59)	68	Transition readiness^at^ Frequency of use Perceived usefulness	D&B: 78.6

^a^RCT: randomized controlled trial.

^b^Self-reported medication adherence was measured using Medication Adherence Report Scale.

^c^Disease control was assessed with the Control of Allergic Rhinitis and Asthma Test, which contains ten questions on asthma and allergic rhinitis symptoms.

^d^Asthma related quality of life was assessed with the Pediatric Asthma Quality of Life Questionnaire.

^e^D&B: Downs and Black checklist.

^f^SRQR: standards for reporting qualitative research.

^g^Self-efficacy was assessed using the Patient Activation Measure.

^h^Self-management skills were assessed using the Transition Readiness Assessment Questionnaire and the University of North Carolina (UNC) TRxANSITION Scale.

^i^Sickle cell disease knowledge was assessed using a 25-item disease-specific knowledge questionnaire.

^j^Health motivation was assessed using the Treatment Self-Regulation Questionnaire.

^k^Health-related quality of life was assessed using the PedsQL (Pediatric Quality of Life Inventory Sickle Cell Disease Module).

^l^Knowledge assessment is paper-based and comprises 12 multiple-choice questions of the adolescent sickle cell disease-specific knowledge.

^m^Self-management confidence was assessed with the Self-Management Skills Checklist.

^n^Not available.

^o^Engagement in the mobile app was measured by (1) dividing the number of log-ins by the number of days with access to the app (eg, log-ins or access days) and (2) the calculating number of completed self-management goals.

^p^Self-efficacy skills was measured by the Patient Activation Measure.

^q^Adherence was measured by the Diabetes Behavior Rating Scale.

^r^Quality of life was self-reported by the adolescent using the PedsQL (Pediatric Quality of Life Inventory Sickle Cell Disease Module) generic scale.

^s^Family conflict was measured using the Revised Diabetes Family Conflict Scale.

^t^Satisfaction with the app was measured by using 8 questions from the Post-Study System Usability Questionnaire.

^u^Usage was measured by the number of days used, a day of usage was counted if the child entered the app, and the number of blood glucose entries per day and the number of messages sent per day were also considered.

^v^A repeat venipuncture for HbA_1c_ levels was performed at a laboratory after the intervention.

^w^Asthma control was measured by the Asthma Control Questionnaire.

^x^Asthma quality of life was measured using the Mini Asthma Quality of Life Questionnaire.

^y^Usefulness, satisfaction, and acceptability of the app were measured by the self-made questionnaire.

^z^The contribution of the app to asthma self-management was measured by the self-made questionnaire.

^aa^Use of roadmap was calculated by ratio variables to account for the differential length of access to Roadmap (version 1.0; Pi Network) across participants.

^ab^Global distress was measured using Profile of Mood States Second Edition, a 65-item measure.

^ac^Family asthma management was measured with the Family Asthma Management System Scale.

^ad^Asthma control was assessed using the asthma control test.

^ae^Asthma management self-efficacy was assessed with the Asthma Management Efficacy Questionnaire.

^af^Family communication was measured using the joint decision-making subscale of the Decision-Making Involvement Scale.

^ag^Medication adherence was measured by a 3-item visual analog scale.

^ah^Adherence motivation was measured by the Rollnick Readiness Ruler.

^ai^Immunosuppressant level was measured by laboratory blood draws to measure trough levels of immunosuppressant medications.

^aj^Usability testing using a research protocol.

^ak^System usability scale was measured by a system usability scale that is a simple, reliable, 10-item scale.

^al^Medication adherence was assessed by app-monitored registrations of Tac dose and time of dosing, measurement of trough Tac variability before, during, and after the study period, and patient self-report (BAASIS [Basel Assessment of Adherence to Immunosuppressive Medication Scale]-questionnaire and interview).

^am^Adherence was assessed by pill counts, pill counts of all inflammatory bowel disease medication prescribed to the patient were completed by patients or caregivers via the portal at assessment time points.

^an^Quality of life was assessed by PedsQL (Pediatric Quality of Life Inventory 4.0).

^ao^Patient-reported symptoms was assessed by the partial Harvey Bradshaw Index.

^ap^User interaction satisfaction was assessed by Questionnaire for User Interaction Satisfaction after engaging with the app for 4 weeks.

^aq^Heuristic evaluation included a display of system status; degree of correspondence with the real world; user control freedom; consistency; fool-proof and error-proof mechanisms; app problem resolution using cognition instead of memory; flexibility and efficiency of use; design aesthetics; error detection, debugging, and recovery from errors; and appropriate assistance and instructions.

^ar^Think-aloud evaluation included asking the users to speak loudly about (1) how to operate the app, (2) why the operation is completed this way, and (3) how they feel about it.

^as^Asthma Control Test questionnaire, each answer to a question is scored between 1 and 5, with the total score ranging from 5 to 25.

^at^Transition readiness was assessed by the Transition-Questionnaire.

### Study Design

Among the included studies, 12 involved an intervention efficacy trial. A randomized controlled trial was the most commonly used design (n=4) [31,33,37,52], followed by a pilot randomized controlled trial (n=3) [32,43,44] and a pretest-posttest design (n=3) [38,47,51]. The remaining intervention efficacy trials used a single-arm open-label study design [48] or a nonrandomized controlled study design [41].

A total of 9 studies used a qualitative approach: 1 multiphase study that involved the iterative design and development of an intervention [32], 1 feasibility study [49], 1 usability study involving an intervention [35], 2 evaluations of user experiences [36,42], and 4 mixed methods studies [39,45,46,50]. In addition, 6 studies included a questionnaire component in the study design: 1 feasibility study [40], 1 evaluation of user engagement [34], and 4 mixed methods studies [39,45,46,50].

### Quality Assessment

An evaluation of the comprehensiveness of reporting was also conducted for all studies. All mixed methods studies were assessed using 2 separate checklists [39,45,46,50]. One study, which was identified by the authors as using a mixed methods approach, involved multiple methods, including a participatory workshop, individual interviews, and user evaluations [45]. Overall, the reporting of questionnaires received considerably lower ratings (n=6; scores were derived using the questionnaire developed by Tong et al [29]: 4/14-9/14, with a scoring rate of 28.6%-64.3%) than the intervention efficacy trials (n=12; Downs and Black scores: 12/28-23/28, with a scoring rate of 42.9%-82.1%) or qualitative studies (n=9; SRQR scores: 9.5/21-17.5/21, with a scoring rate of 45.2%-83.3%).

Among the 12 studies that reported an intervention efficacy trial [31-33,37,38,41,43,44,47,48,51,52], 91.7% (11/12) were fair-to-good-quality studies [31-33,37,38,41,43,44,47,51,52] (Multimedia Appendix 3). Among the 9 qualitative research papers [32,35,36,39,42,45,46,49,50], 4 were missing at least 40% of the items that were identified as important on the SRQR checklist (Multimedia Appendix 4). All 6 studies that included a questionnaire were missing at least 40% of the items that were identified as important with the 16-item checklist of Tong et al [29] for reporting questionnaire studies [34,39,40,45,46,50] (Multimedia Appendix 5).

### Summary of Interventions

Among the 22 studies that described the intervention (Multimedia Appendix 6), 12 were evaluated in the context of an efficacy trial. All 12 evaluations focused on web-based interventions. Most interventions were delivered via a mobile app (n=18) [31-33,35-40,42,43,45-47,49-52]. Other modes of delivery included websites (n=2) [34,48] and telephones (n=2) [41,44]. In addition, only 27.3% (6/22) of the interventions were based upon extant theories [32,35,39,42,46,50], such as goal-setting theory [53], self-determination theory [54], social cognitive theory [55], and transition theory [56]. The interventions were summarized into 6 themes: medication monitoring and reminders, symptom tracking and monitoring, management goal setting, knowledge education and self-management skills training, incentives and reinforcement, and communication.

### Theme 1: Medication Monitoring and Reminders

A total of 12 interventions focused on medication monitoring and reminders that targeted multiple aspects of nonadherent behavior, such as medication reminder or alarm [31,32,36,39,42,46], medication usage tracking [35,42,45,47,52], evaluations of medication adherence [31,43,47,48], positive feedback messages [42]. Two good-quality studies described the intervention designed to improve medication adherence. The “ADAPT” (Adolescent Adherence Patient Tool) app was securely connected to a desktop app connected to the patient’s own community pharmacist, which provided functions of medication reminder alarms, chats with the pharmacist, and two questions that were answered once every two weeks to monitor nonadherence [31]. TusenTac (University of Oslo) is a mobile app that features a tailored design for solid organ transplant recipients. In addition to serving as an immunosuppressive medication monitor, it offers the functions of medication reminder alerts and evaluations of medication adherence [47].

### Theme 2: Symptom Tracking and Monitoring

The 9 interventions in this review involved symptom tracking and monitoring [31,33,35-37,43,45,50,52]. Specifically, the functions include asthma symptoms assessment and tracking [31,35,36,43,45,52], asthma status feedback [36], blood glucose levels tracking and reminders [50], and daily pain and mood symptoms assessment [33,37]. One of the good-quality interventions focused on tracking and monitoring symptoms based on a real time ecological momentary assessment of asthma symptoms [35]. In another fair-quality study, iManage was used to record progress per the daily pain and mood symptoms exhibited by adolescents with sickle cell disease [37].

### Theme 3: Management Goal Setting

The 4 apps included in this review featured self-management goal setting [33,37,39,43]. Two studies focused on asthma patients [33,37], while the other two focused on sickle cell disease patients [39,43]. Two fair-quality studies described the interventions to promote self-management goal setting. AIM2ACT helped adolescents and caregivers identify an asthma management goal after a 1-week period focusing on the assessment of needs. Dyads were then guided through the process of behavioral contracting to outline the specific steps that each person would take to achieve the goal, how long the goal would take to complete, and the reward for accomplishing the goal [43]. iManage is a mobile app that users can create, monitor, and complete self-management goals (eg, exercising, taking medications, or sleeping). They could also link sickle cell disease symptoms to their goals in a visual calendar and see the progress of other users (eg, goals complete or not completed) [37].

### Theme 4: Knowledge Education and Self-Management Skills Training

Ten of the interventions provided knowledge education and skills training [31-35,39,43,46,50,51]. The content of information and skills training includes disease-related information [31-33,39,46,50,51], information concerning specific topics of interest to adolescents (such as career development, sex, or pregnancy) [50], transition knowledge and skills [34], asynchronous inhaler use technique [35], and communication trainings with caregivers on asthma management needs [43]. The modes of delivery include movies [31], videos [34,35,43,51], e-books [39,50], phone calls [33], text information [32,51], and websites [46]. One good-quality study described short educational and motivational movies on asthma-related topics [31]. In another fair-quality study, AIM2ACT is a dyadic mHealth intervention providing separate and tailored skills-training videos for adolescents and caregivers (eg, how adolescents can effectively communicate their asthma management needs to caregivers [43].

### Theme 5: Incentives and Reinforcement

There are a total of 6 of the interventions that provided rewards to encourage and reinforce self-management behaviors [32,34,38,42,47,50]. The forms of rewards include point rewards [32,38,50], small toys [34], and gamification features [34,42,47]. Two fair-to-good-quality studies described incentive interventions to stimulate continuous use. Users of the “MyT1DHero” app could redeem points for accessories for their “hero” avatar on the app, including capes, boots, logos, masks, and different hair colors and styles [38]. The TusenTac app was age-adapted by including different fun facts that appeared after medication registration; furthermore, it featured a tailored “transplant-designed” gamification system with challenges [47]. A total of 4 of the interventions used cognitive strategies to promote behavioral activation included motivational interviewing [35,37], cognitive behavioral strategies [37,39], and praise text messages [44]. One good-quality study provided 6 weekly, 90-minute group sessions guided by psychologists, which included culturally sensitive motivational interviewing and cognitive behavioral strategies with the goal of enhancing behavioral activation [37]. In another good-quality study, Sayegh et al [44] provided text messages containing praise to adolescents whose laboratory tests indicated immunosuppressant medications within the expected range with the goal of improving their medication adherence.

### Theme 6: Communication

A total of 7 mobile apps included the feature to facilitate social media interactions with parents, peers, and health care providers [31,33,38,42,45,50,51]. In one good-quality study, the ADAPT app provided functions of peer chat and pharmacist chat functions to facilitate contact [31]. In another fair-quality study, the MyT1DHero app focused on parent-child interactions to promote positive communication regarding T1D management through 2 separate app interfaces—one for the adolescent and one for the parent. In addition, there are also peer support functions by providing videos of other adolescents with T1D telling their stories and affirming messages [38].

### Outcomes

Study outcomes varied based on the stage of development (feasibility, usability, efficiency, and effectiveness) of the intervention in question. The usability outcomes and health outcomes of this research were summarized per the aim of each intervention (Multimedia Appendices 7-10).

### Perceived Usability or Acceptability

Perceived usability was explored in the context of 11 interventions [32,35,36,39,40,43,45,48-50,52]. Most of these interventions (n=10) [32,35,36,39,43,45,48-50,52] were evaluated positively by the participants. Most participants felt that the apps were acceptable because they were convenient, easy to use, and easy to access when they needed help managing their health [32,35,36,39,43,45,49,50,52].

### User Engagement

The overall levels of user engagement during the intervention were variable [32,35,39,40,43,48,52]. Schwartz et al [32] found that youth engagement with the app ranged from 19.3% to 98.2% of app intervention days, with a median of 63.9% of days actively using the app. Davis et al [39] found that over 6 weeks, without follow-up appointments, telephone calls, or reminders from the research staff, 33% (4/12) of participants used the app one to five times, 25% (3/12) participants used it six to ten times, and 16.7% (2/12) participants used it more than 10 times. Some studies indicated a gradual decline in youth engagement with these apps over time [40,52]. Brookshire-Gay et al [40] showed that, among users (n=22), engagement rates with Roadmap (version 1.0) were 96% and 91% for the first 2 weeks, respectively; however, this percentage declined to 58% (n=12) by week 4. Han et al [52] reported that only 30%-66% of participants either sometimes or frequently used the app at the 3-month mark. Some studies identified barriers preventing user engagement, including time-consuming video uploads [35], noncontinuous access to a phone, literacy levels, limited free time [36], font size, language, and false impressions [49].

### Preferred Delivery Method

Adolescents and young adults’ preferred delivery method involved visually appealing features [35,36,39,49,50]. For example, colors, backgrounds, graphics, and fun or entertaining elements, such as games, avatars, incentives, design elements, and additional customization options, encouraged them to use these apps more frequently and to be more engaged with the apps. Schneider et al [36] found that many adolescents and young adults appreciated the options to change the wallpaper color and designs on the page and to customize the “emoji” faces they used to express how they felt at the time of data entry.

### Preferred Features

Suggestions regarding the inclusion of disease-specific information were made by adolescents and young adults [35,39]. For example, patients with asthma mentioned information concerning strategies to support the self-management of asthma, general information about asthma, and role model (celebrity or athletes) testimonials concerning how they coped with asthma. Communication was mentioned at several levels [35,36], including communication with peers, health care providers, parents, and app teams. Many adolescents and young adults reported that they would appreciate the opportunity to connect with their peers and share their feelings via social media, while some adolescents and young adults appropriated being able to contact or share information with their health care providers [35,36]. Although the use of technology, whether through messaging or video interactions, was strongly preferred by most adolescents and young adults as their primary means of routine communication and interaction, none of the participants wanted to completely replace traditional face-to-face encounters [35].

### Health Outcomes

#### Symptom Control

Three fair-to-good-quality studies reported symptom control assessments, which focused on adolescents and young adults with asthma (aged 12-22 years) [31,43,51]. One fair-quality study conducted asthma control assessments at baseline, postintervention, and 4-month follow-up. Results showed that participants randomized to the intervention group (AIM2ACT) had significant improvements (*P*=.04) in asthma control scores compared to the control group [43]. Another fair-quality study revealed that there was a significant difference (*P*<.001) in the pretest and posttest scores of the asthma control score from the intervention group, while no difference was found compared to the control group [51]. In contrast to these above results, one good-quality study [31] found that after six months of access to the ADAPT intervention, no effect was observed on asthma control compared to the control group (*P*>.05).

#### Self-Reported Medication Adherence

Four fair-to-good-quality studies reported medication adherence [31,38,44,47] through self-report questionnaires [31,38,47], the medication level variability index [44,47], as well as app-monitored registrations of medication dose and time of dosing [47]. Two good-quality studies reported that mHealth-based intervention had a positive effect on medication adherence compared to the control group (*P*<.05) [31,44]. Different from the above results, two fair-to-good-quality preclinical or postclinical trials demonstrated that there was no significant change in medication adherence after the short-term intervention (*P*>.05) [38,47]. In addition, Kindem et al [47] found that four of eleven (36%) who were nonadherent assessed at inclusion turned adherent during the intervention period, and after the intervention, 70% reported improved timing-adherence at the interview.

#### Quality of Life

Among the five studies to focus on this topic, three (67%) fair-to-good-quality studies reported on quality of life [31,38,43]. Among these 3 studies, 2 (75%) focused on adolescents and young adults with asthma (aged 12-18 years). One fair-quality study [43] found participants randomized to the AIM2ACT cohort had significant improvements in asthma-related quality of life at postintervention (*P*=.002) and at the 4-month follow-up (*P*=.002) compared to control that surpassed the minimally clinically important difference threshold. However, the good-quality study conducted by Kosse et al [31] reported that compared to the control group, no intervention effect was found on asthma-related quality of life at baseline and at the 6-month follow-up (*P*>.05). In a fair-quality preclinical or postclinical trial, significant benefits were demonstrated in quality of life of adolescents and young adults with type 1 diabetes (*P*=.001) after 12 weeks of the MyT1DHero app intervention [38].

#### Disease Knowledge

Two studies reported on disease knowledge [33,34], which was measured using questionnaires. Saulsberry et al [34] found that there was a positive correlation between participation rate of Sickle Cell Transition E-Learning Program (STEP) intervention (6-module tool) and disease knowledge scores (*P*=.003), and participants who completed ≥3 STEP modules had higher disease knowledge scores compared with those who completed <3 STEP modules (*P*=.007) [34]. However, another good-quality randomized controlled trial showed that although the knowledge scores of participants significantly increased compared to baseline after 6 weeks of SCThrive (sickle cell disease self-management intervention) intervention (*P*<.001), there was no statistically significant difference compared to the control group (*P*>.05) [33].

#### Self-Management Development

Four studies reported on self-management development [33,34,37,43], which was measured in terms of self-management confidence [34], self-management skills [33,37], and self-management efficacy [33,37,43]. One fair-quality randomized controlled trial [37] found that the number of logins to the mobile app significantly predicted adolescents and young adults-reported self-management skills (*P*<.05), while no correlation was found in the retrospective cohort study [34] between the number of modules completed and the self-management confidence. One fair-quality study [43] indicated that compared to the control group, no intervention effect was found on self-efficacy at baseline and at the 4-month follow-up (*P*>.05), consistent with research findings of the good-quality study conducted by Crosby et al [33]. Moreover, Crosby et al [33] found that although no significant change was found in overall self-management after SCThrive intervention (*P*>.05), there was statistically significant improvement in one self-management skill (tracking health; *P*=.001).

## Discussion

### Principal Findings

This systematic review examined 22 articles that were published between 2019 and 2023 and focused on adolescents and young adults aged 9 to 25 years. Despite the proliferation of eHealth and mHealth interventions during the past 5 years [57], the overall evidence regarding the efficacy, cost-effectiveness, and long-term health benefits of these interventions is minimal, as most of the interventions identified were in the usability testing stage.

Using the qualitative data, this systematic review revealed most adolescents and young adults felt the interventions delivered by mobile apps or websites were feasible, acceptable, and useful, as they were convenient and easy to use in the context of helping adolescents and young adults manage their health [32,35,36,39,43,45,49,50,52]. Low and Manias [24] confirmed that adolescents and young adults were receptive to receiving health information electronically. In fact, multiple studies have shown that most adolescents and young adults have access to mobile phones that they keep with them at all times and they are interested in using mHealth and apps to manage their health and facilitate the health care transition [58,59]. However, engaging adolescents and young adults in research could be difficult. In this system review, we found that the overall levels of engagement during the intervention were variable [32,35,39,40,43,48,52] and tended to decrease over time [40,52]. Some adolescents and young adults could be defined as “super users” who logged into the app nearly every day, while others engaged with the app infrequently [37,39]. Previous studies have shown that early involvement promotes better engagement [60,61], and when making choices among many apps, users prefer certain features tailored to their needs and preferences [62,63]. This finding highlights the importance of stakeholder involvement in the design process, as adolescents are undergoing a critical period concerning the development of routines and self-management skills, and digital interventions designed for adults may not address the unique developmental and psychosocial barriers faced by adolescents [64]. In addition, a previous study indicated that increasing caregiver involvement can effectively improve the engagement of adolescents and young adults in digital interventions [65]. Considering the high risk that adolescents and young adults will not comply with the intervention, allowing them to designate people other than their caregivers (ie, older siblings or friends) to increase accountability may help them assert their independence from their caregivers.

Adolescents and young adults had different preferred styles of message delivery and functions. Specifically, adolescents and young adults have partiality for more engaging elements (such as visually appealing features, customization options, or fun or entertaining functions) [35,36,39,49,50], disease-specific information [35,39], and opportunities to communicate with their peers, health care providers, and app teams [35,36]. Education is an important aspect of the task of promoting the development of self-management skills [66]. The continuous highlighting of disease-specific educational themes via a web-based device using video or text can enable patients to easily access reliable information. Interacting with peers and health care providers via social media may promote better outcomes [67]. Adolescents and young adults would prefer to network with their peers, which would enable them to share their experiences and offer each other emotional and informational support. Previous studies have proved that face-to-face support groups and peer guidance were underused but highly anticipated resources among adolescents and young adults with chronic diseases, which should be fully explored in the self-management and health care transition in the future [68]. In addition, some adolescents and young adults preferred to share information or discuss their concerns with their health care providers. Dwyer-Matzky et al [69] found that 59% of adolescents and young adults with chronic diseases would like to establish close connections with medical teams, which has been proven to effectively improve treatment adherence.

From the perspective of intervention effectiveness, although digital health interventions have great potential in the context of adolescent disease management, some results have indicated insufficient evidence of improvement in terms of adolescent self-management behavior and biomedical outcomes. For example, some fair-to-good-quality randomized controlled trials showed that compared to the control group, there were no significant improvements in asthma control scores [31], quality of life [31], disease knowledge [33], and self-efficacy [33,43] at postintervention (*P*＞.05). A meta-analysis conducted by Low and Manias [24] did not reveal any differences in the impact of interventions provided through mobile apps and websites on quality of life, self-management, which could mainly be attributed to the lack of high-quality randomized controlled trials. Due to the small number and heterogeneity of the studies, meta-analysis was precluded in this study. Therefore, we cannot draw a conclusion about the efficacy of the eHealth and mHealth interventions on symptom control, self-reported medication adherence, quality of life, disease knowledge, and self-management development from this review. Future large-scale, high-quality randomized controlled trials and investigations of the efficacy and sustainability of eHealth and mHealth interventions should be conducted. In addition, among the included studies, only one study [52] focused on the health care transition process and evaluated transition readiness. In the systematic review conducted by Pérez et al [23], none of the studies measured the transition process as an outcome. Evaluating transition readiness has been widely regarded as an important component of optimizing transition outcomes due to its ability to identify obstacles to transition, individually plan for treatment, and monitor progress over time [70]. More attention should be paid to the transitional outcomes especially transition readiness of adolescents and young adults with chronic diseases to facilitate smooth health care transition and optimize patient health outcomes.

From a cost-effectiveness perspective, eHealth and mHealth technologies may exhibit greater cost-effectiveness than face-to-face consultation or clinical medical care. Due to its ability to overcome spatial limitations, this approach can greatly reduce the time and transportation costs for both users and professional medical personnel [71]. Nevertheless, little is known about the cost-effectiveness of developing and maintaining such technology-based interventions. The World Health Organization strongly recommends that cost analyses of interventions should be conducted [72]. Relevant costs include long-term direct and indirect costs, ranging from digital health intervention development to training and implementation, as well as the ultimate benefits for patients and health care systems, such as improving patient health outcomes and reducing human resource costs. However, no studies included in this review evaluated the economic characteristics and cost-effectiveness of digital interventions, while only one study made a reference to cost in the review by Pérez et al [23]. Two systematic reviews indicated a lack of economic data to support eHealth and mHealth interventions among adolescents and young adults with chronic diseases [22,73]. Butler et al [22] suggested considering costs as early as possible during the process of prototype development to help make strategic decisions, while Badawy et al [73] highlighted the need for a comprehensive economic evaluation of technology-based interventions to facilitate more evidence-based assessments of the scalability, sustainability, and benefits of broader investment of such technology tools among adolescents and young adults with chronic diseases.

### Limitations

This review has several limitations. First, our search strategy was limited to an academic context, and we focused on electronically indexed health databases published in peer-reviewed journals rather than apps contained in commercial stores. Second, the studies included in this review used quantitative, qualitative, and mixed methods, which made it difficult to compare their quality. As the heterogeneity was observed across different studies and the different stages of intervention development (feasibility, usability, efficiency, and effectiveness) on which they focused, we used a descriptive synthesis-based methodology. This form of analysis can be subjective, and it faces the risk of reporting bias. To mitigate this risk and improve transparency, all the authors reviewed all the stages of the data analysis. Finally, some relevant studies might have been missed because only studies published in English were included. It was acknowledged that a search including foreign language databases may reveal additional studies published in languages other than English in low- and middle-income countries. Given these limitations, the findings of this systematic review should be interpreted with caution.

### Conclusions

This systematic review revealed that adolescents and young adults were receptive to and interested in receiving information and managing their health using a mobile app or website. It should be noted that adolescents and young adults had different preferred styles of message delivery and functions. Therefore, to provide an age-appropriate, reliable condition-specific resource and improve patient engagement during the transition process, the best approach would be to involve adolescents and young adults early in the design process to identify their specific needs and preference. This would be better to be coupled with or followed by obtaining suggestions from health care providers and app teams. As most of the studies remained in the early stages of exploration, there remain limited data about the effectiveness of eHealth and mHealth interventions facilitating self-management and health care transition of adolescents and young adults with chronic diseases. Large sample, multicenter randomized controlled trials should be conducted in the future to verify the effectiveness of eHealth and mHealth interventions. Moreover, future studies could pay more attention to the transitional outcomes (especially transition readiness), and cost-effectiveness of developing and maintaining eHealth and mHealth interventions.

## Appendix

Multimedia Appendix 1 Search strategies.

Multimedia Appendix 2 PRISMA checklist. PRISMA: Preferred Reporting Items for Systematic Reviews and Meta-Analyses.

Multimedia Appendix 3 Quality appraisal of intervention efficacy trials using the Down and Black checklist.

Multimedia Appendix 4 Quality appraisal of the qualitative component of the studies.

Multimedia Appendix 5 Quality appraisal of the questionnaire component of the studies.

Multimedia Appendix 6 Characteristics of included interventions.

Multimedia Appendix 7 A summary of usability study or users’ perceived acceptance of the intervention.

Multimedia Appendix 8 Main outcomes.

Multimedia Appendix 9 Synthesis of qualitative results.

Multimedia Appendix 10 A summary of intervention efficacy evaluations.

## Abbreviations

ADAPT: Adolescent Adherence Patient Tool
mHealth: mobile health
PRISMA: Preferred Reporting Items for Systematic Reviews and Meta-Analyses
PROSPERO: International Prospective Register of Systematic Review
SRQR: standards for reporting qualitative research
STEP: Sickle Cell Transition E-Learning Program
