# Supplementary material for: Usability and Effectiveness of eHealth and mHealth Interventions That Support Self-Management and Health Care Transition in Adolescents and Young Adults With Chronic Disease: Systematic Review
Source: J Med Internet Res. 2024 Nov 26;26:e56556. doi: 10.2196/56556 (PMC11632288; doi:10.2196/56556)
Supplement: Multimedia Appendix 1 [file jmir_v26i1e56556_app1.docx]

| Database | Literature | Search Strategy |
| --- | --- | --- |
| MEDLINE | 2126 | (("adolescent"[MeSH Terms] OR "young adult"[MeSH Terms] OR ((((("adolescen*" [Title/Abstract] OR "young adult*"[Title/Abstract]) OR "young person*"[Title/Abstract]) OR "young people"[Title/Abstract]) OR "teen*"[Title/Abstract]) OR "youth*"[Title/Abstract]) OR "young patient*"[Title/Abstract])))) AND (("[mobile applications](https://www.ncbi.nlm.nih.gov/mesh/68063731)"[MeSH Terms] OR (((("eHealth" [Title/Abstract]) OR "mhealth"[Title/Abstract]) OR "mobile health"[Title/Abstract]) OR "electronic"[Title/Abstract]) OR "phone*"[Title/Abstract]) OR "telehealth"[Title/Abstract]) OR "application*"[Title/Abstract]) OR "app*"[Title/Abstract]) OR "mobile applications"[Title/Abstract]) OR "web"[Title/Abstract]) OR "smartphone*" [Title/Abstract])) OR "technology"[Title/Abstract])))) AND (("self-management"[MeSH Terms] OR "transition to adult care"[MeSH Terms] OR ((((self care" [Title/Abstract] OR "disease management"[Title/Abstract]) OR "medical management"[Title/Abstract]) OR "transition"[Title/Abstract] OR "transitional"[Title/Abstract]))))  Filters: Full text, Humans, English, from 2019 - 2024. |
| Embase | 4627 | (('adolescen*'/exp) OR( 'young adult*' OR 'young person*' OR 'young people' OR 'teen*' OR 'youth*' OR 'young patient*':ti)) AND ('[mobile applications](https://www.ncbi.nlm.nih.gov/mesh/68063731)'/exp OR 'eHealth'/exp OR 'mhealth'/exp OR 'mobile health'/exp OR 'electronic'/exp OR 'phone*'/exp OR 'application*'/exp OR 'app*'/exp OR 'web'/exp OR 'smartphone*'/exp OR 'technology*'/exp OR 'telehealth'/exp) AND ('self-management'/exp OR 'self care'/exp OR 'disease management'/exp OR 'medical management'/exp OR 'transition to adult care'/exp OR 'transition'/exp OR 'transitional'/exp) |
| CINAHL | 1772 | AB(adolescen* OR young adult* OR young person* OR young people OR teen* OR youth* OR young patient*) AND TI ([mobile applications](https://www.ncbi.nlm.nih.gov/mesh/68063731) OR eHealth OR mhealth OR mobile health OR electronic OR phone* OR application* OR app* OR web OR smartphone* OR technology* OR telehealth) AND AB(self-management OR self care OR disease management OR medical management OR transition to adult care OR transition OR transitional) |
| ProQuest | 4668 | ab (adolescen* OR young adult* OR young person* OR young people OR teen* OR youth* OR young patient*) AND ti([mobile applications](https://www.ncbi.nlm.nih.gov/mesh/68063731) OR eHealth OR mhealth OR mobile health OR electronic OR phone* OR application* OR app* OR web OR smartphone* OR technology* OR telehealth) AND ab(self-management OR self care OR disease management OR medical management OR transition to adult care OR transition OR transitional) filter:full-text, English, from 2019 - 2024. |
| Web of Science | 3559 | (MeSH descriptor: [adolescent] explode all trees or (adolescen* or young adult* or young person* or young people or teen* or youth* or young patient*)):ti,ab,kw and (MeSH descriptor: [mobile application](https://www.ncbi.nlm.nih.gov/mesh/68063731)] explode all trees or (eHealth or mhealth or mobile health or electronic or phone* or application* or app* or web or smartphone* or technology* or telehealth)):ti,ab,kw and (MeSH descriptor: transition to adult care] explode all trees or (transition or transitional or self-management or self care or disease management or medical management)):ti,ab,kw |
