# Supplementary material for: Usability and Effectiveness of eHealth and mHealth Interventions That Support Self-Management and Health Care Transition in Adolescents and Young Adults With Chronic Disease: Systematic Review
Source: J Med Internet Res. 2024 Nov 26;26:e56556. doi: 10.2196/56556 (PMC11632288; doi:10.2196/56556)
Supplement: Multimedia Appendix 2 [file jmir_v26i1e56556_app2.docx]

| **Section and Topic** | **Item #** | **Checklist item** | **Location where item is reported** |
| --- | --- | --- | --- |
| **TITLE** | | |  |
| Title | 1 | Identify the report as a systematic review. | 1 |
| **ABSTRACT** | | |  |
| Abstract | 2 | The abstracts include Background, Objective, Design, Methods, Results and Conclusions. | Title page |
| **INTRODUCTION** | | |  |
| Rationale | 3 | Describe the rationale for the review in the context of existing knowledge. | 7-8 |
| Objectives | 4 | Provide an explicit statement of the objective(s) or question(s) the review addresses. | 8 |
| **METHODS** | | |  |
| Eligibility criteria | 5 | Specify the inclusion and exclusion criteria for the review. | 9-10 |
| Information sources | 6 | Specify all databases, registers, websites, organisations, reference lists and other sources searched or consulted to identify studies. Specify the date when each source was last searched or consulted. | 9 |
| Search strategy | 7 | Present the full search strategies for all databases, registers and websites, including any filters and limits used. | 9 |
| Selection process | 8 | Search results were imported into Endnote X9 software and filtered to eliminate deduplicates. Each of the two reviewers (ZRL and JYW) independently screened studies by the titles and abstracts to confirm whether the study met the inclusion criteria. Then, two reviewers read the full texts of the selected studies in detail and recorded the reasons for exclusion of full-text papers. In all cases, the decision to include or exclude a single study was approved by both reviewers. | 10-11 |
| Data collection process | 9 | Data were extracted independently by two reviewers.The final extracted data were reviewed and discussed by the research team. | 11 |
| Data items | 10a | We developed a standardized form (including author name of the first author, year and country, age, gender and chronic condition, study design, sample size, duration, intervention media, intervention components and quality assessment and study outcomes) to extract the data. If any discrepancies between the reviewers emerged, they were resolved through discussion with the wider research team. | 11 |
|  | 10b | We have listed and defined all other variables for which data were sought . There were no any missing or unclear information. | 11 |
| Study risk of bias assessment | 11 | Not applicable. |  |
| Effect measures | 12 | The Downs and Black (D&B) checklist for randomized and nonrandomized studies was used to appraise the quality of the intervention efficacy trials; The Standards for Reporting Qualitative Research (SRQR) checklist was used for qualitative research (O Brien et al., 2014), and a 16-item tool was used for questionnaire research. | 11-12 |
| Synthesis methods | 13a | Due to the heterogeneity exhibited by different studies and the different stages of intervention development, meta-analysis was deemed unsuitable for this systematic review. Instead, we used a narrative synthesis methodology to organize, explore, and present potential similarities and differences, associations, and patterns of data across different studies. | 12 |
|  | 13b | For qualitative research, including exploratory and feasibility studies, mixed methods studies, and studies that involved interviews o focus groups, NVivo software (version 11; QSR International Pty Ltd.) was used to extract and organize the data. The three-stage thematic synthesis outlined by Thomas and Harden was applied to the data synthesis and thematic analysis by one reviewer. | 12 |
|  | 13c |  |  |
|  | 13d |  |  |
|  | 13e |  |  |
|  | 13f |  |  |
| Reporting bias assessment | 14 | Not applicable.. |  |
| Certainty assessment | 15 | Disagreements were resolved through discussion and, if necessary, evaluation by a third reviewer. | 12 |
| **RESULTS** | | |  |
| Study selection | 16a | We described the results of the search and selection process, from the number of records identified in the search to the number of studies included in the review, and we usied a flow diagram. | 12 |
|  | 16b | Not applicable. |  |
| Study characteristics | 17 | We have cited each included study and present its characteristics. | 13-14 |
| Risk of bias in studies | 18 | Not applicable. |  |
| Results of individual studies | 19 | A brief summary of each study characteristics is outlined inTable 2. | Table 2 |
| Results of syntheses | 20a | For each synthesis, we briefly summarise the characteristics among contributing studies. | Table 2 |
|  | 20b | Present results of all syntheses conducted. |  |
|  | 20c | Not applicable. |  |
|  | 20d | Not applicable. |  |
| Reporting biases | 21 | Not applicable |  |
| Certainty of evidence | 22 | Not applicable. |  |
| **DISCUSSION** | | |  |
| Discussion | 23a | We have provided a general interpretation of the results in the context of other evidence. | 26-30 |
|  | 23b | We have discussed any limitations of the review. | 31 |
|  | 23c | We have discussed any limitations of the review processes used. | 31 |
|  | 23d | We have discussed implications of the results for practice, policy, and future research. | 32 |
| **OTHER INFORMATION** | | |  |
| Registration and protocol | 24a | We have provided registration information for the review, including register name and registration number. | 9 |
|  | 24b | Not applicable. |  |
|  | 24c | Not applicable. |  |
| Support | 25 | We have described sources of financial support for the review. | 33 |
| Competing interests | 26 | We have declared any competing interests of review authors. | 33 |
| Availability of data, code and other materials | 27 | We don’t report which of the following are publicly available and where they can be found. | 33 |

*From:*  Page MJ, McKenzie JE, Bossuyt PM, Boutron I, Hoffmann TC, Mulrow CD, et al. The PRISMA 2020 statement: an updated guideline for reporting systematic reviews. BMJ 2021;372:n71. doi: 10.1136/bmj.n71

For more information, visit: <http://www.prisma-statement.org/>
