# Supplementary material for: Usability and Effectiveness of eHealth and mHealth Interventions That Support Self-Management and Health Care Transition in Adolescents and Young Adults With Chronic Disease: Systematic Review
Source: J Med Internet Res. 2024 Nov 26;26:e56556. doi: 10.2196/56556 (PMC11632288; doi:10.2196/56556)
Supplement: Multimedia Appendix 3 [file jmir_v26i1e56556_app3.docx]

| **Item** | **[31]** | **[32]** | **[33]** | **[37]** | **[38]** | **[41]** | **[43]** | **[44]** | **[47]** | **[48]** | **[51]** | **[52]** |
| --- | --- | --- | --- | --- | --- | --- | --- | --- | --- | --- | --- | --- |
| **Reporting** | | | | | | | | | | | | |
| 1 | Yes | Yes | Yes | Yes | Yes | Yes | Yes | Yes | Yes | Yes | Yes | Yes |
| 2 | Yes | Yes | Yes | Yes | Yes | Yes | Yes | Yes | Yes | Yes | Yes | Yes |
| 3 | Yes | Yes | Yes | Yes | Yes | Yes | Yes | Yes | Yes | Yes | Yes | Yes |
| 4 | Yes | Yes | Yes | Yes | Yes | Yes | Yes | Yes | Yes | Yes | Yes | Yes |
| 5 | Yes | No | Yes | No | Partially | Yes | Partially | Yes | Partially | No | Partially | Yes |
| 6 | Yes | Yes | Yes | Yes | Yes | Yes | Yes | Yes | Yes | No | Yes | Yes |
| 7 | Yes | Yes | Yes | Yes | Yes | Yes | Yes | Yes | Yes | No | Yes | Yes |
| 8 | No | No | No | No | No | No | No | No | Yes | No | No | No |
| 9 | Yes | Yes | Yes | Yes | Yes | Yes | Yes | Yes | Yes | Yes | Unable to  Determine | Yes |
| 10 | Yes | Yes | Yes | Yes | Yes | No | Yes | Yes | Yes | Yes | Yes | No |
| **External validity** | | | | | | | | | | | | |
| 11 | Yes | No | Yes | No | No | Yes | Unable to  Determine | Yes | Unable to  Determine | Unable to  Determine | Unable to  Determine | Yes |
| 12 | Yes | No | Yes | No | Unable to  Determine | Yes | Unable to  Determine | Unable to  Determine | Unable to  Determine | Unable to  Determine | Unable to  Determine | Yes |
| 13 | Unable to  Determine | Unable to  Determine | Unable to  Determine | Unable to  Determine | Unable to  Determine | Yes | Unable to  Determine | Unable to  Determine | Unable to  Determine | Unable to  Determine | Unable to  Determine | Unable to  Determine |
| **Internal validity - bias** | | | | | | | | | | | | |
| 14 | No | No | No | No | No | No | No | No | No | No | No | Yes |
| 15 | Unable to  Determine | Unable to  Determine | Unable to  Determine | Unable to  Determine | No | No | Unable to  Determine | Yes | No | No | Unable to  Determine | Unable to  Determine |
| 16 | Yes | Yes | Yes | Yes | Yes | Yes | Yes | Yes | Yes | Yes | Yes | Yes |
| 17 | Yes | Unable to  Determine | Yes | Unable to  Determine | Yes | Yes | Yes | Yes | Yes | Yes | Yes | Yes |
| 18 | Yes | Yes | Yes | Yes | Yes | Yes | Yes | Yes | Yes | Yes | Yes | Yes |
| 19 | Yes | Yes | Yes | Yes | Yes | Yes | Yes | Yes | Yes | Unable to  Determine | Unable to  Determine | Yes |
| 20 | Yes | Yes | Yes | Yes | Yes | Yes | Yes | Yes | Yes | Yes | Yes | Yes |
| **Internal validity – confounding (selection bias)** | | | | | | | | | | | | |
| 21 | No | Yes | Yes | Yes | Yes | Yes | No | Yes | Yes | Unable to  Determine | Unable to  Determine | Yes |
| 22 | Yes | Unable to  Determine | Unable to  Determine | Unable to  Determine | Yes | Yes | Yes | Yes | Yes | Yes | Yes | Yes |
| 23 | No | No | Yes | Unable to  Determine | No | No | Unable to  Determine | Yes | No | No | Unable to  Determine | Unable to  Determine |
| 24 | No | No | No | No | No | No | No | No | No | No | No | Unable to  Determine |
| 25 | Yes | Yes | Yes | Unable to  Determine | Unable to  Determine | No | Unable to  Determine | Yes | Unable to  Determine | Unable to  Determine | Unable to  Determine | Yes |
| 26 | Yes | Unable to  Determine | Unable to  Determine | Yes | No | Unable to  Determine | Yes | Yes | Yes | Unable to  Determine | Yes | Yes |
| **Power** | | | | | | | | | | | | |
| 27 | Unable to  Determine | Unable to  Determine | Yes | Yes | Yes | Yes | Yes | Yes | Yes | Yes | Yes | Yes |
| Score* | 20 | 14 | 21 | 15 | 17 | 20 | 17 | 23 | 19 | 12 | 15 | 22 |
| Judgement | good | fair | good | fair | fair | good | fair | good | good | poor | fair | good |

***Higher score indicates higher quality, with yes=1, no=0 and unable to determine=0**

**Total scores: Excellent: 24-28, good: 19-23, fair: 14-18 fair, poor: less than 14 [27]**
