# Supplementary material for: Usability and Effectiveness of eHealth and mHealth Interventions That Support Self-Management and Health Care Transition in Adolescents and Young Adults With Chronic Disease: Systematic Review
Source: J Med Internet Res. 2024 Nov 26;26:e56556. doi: 10.2196/56556 (PMC11632288; doi:10.2196/56556)
Supplement: Multimedia Appendix 4 [file jmir_v26i1e56556_app4.docx]

| **Item** | **[32]** | **[35]** | **[36]** | **[39]** | **[42]** | **[45]** | **[46]** | **[49]** | **[50]** |
| --- | --- | --- | --- | --- | --- | --- | --- | --- | --- |
| **Title and abstract** | | | | | | | | | |
| 1 | 0.5 | 1 | 1 | 0.5 | 0.5 | 0.5 | 0.5 | 1 | 0.5 |
| 2 | 1 | 1 | 1 | 1 | 1 | 1 | 1 | 1 | 1 |
| **Introduction** | | | | | | | | | |
| 3 | 1 | 1 | 1 | 1 | 1 | 1 | 1 | 1 | 1 |
| 4 | 1 | 1 | 1 | 1 | 1 | 1 | 1 | 1 | 1 |
| **Methods** | | | | | | | | | |
| 5 | 0 | 0 | 0 | 0 | 0 | 0 | 0 | 0 | 0 |
| 6 | 0 | 0.5 | 0 | 0 | 0.5 | 1 | 1 | 0 | 1 |
| 7 | 0 | 1 | 0 | 0 | 1 | 0 | 0 | 1 | 1 |
| 8 | 0.5 | 0.5 | 0.5 | 0 | 0.5 | 1 | 0.5 | 0.5 | 0 |
| 9 | 0 | 1 | 1 | 1 | 1 | 1 | 1 | 1 | 1 |
| 10 | 0.5 | 0.5 | 1 | 0 | 0.5 | 0.5 | 0.5 | 1 | 0 |
| 11 | 1 | 1 | 1 | 0 | 1 | 1 | 0.5 | 1 | 0 |
| 12 | 1 | 1 | 1 | 0 | 1 | 0.5 | 0.5 | 1 | 0 |
| 13 | 0.5 | 1 | 1 | 0 | 1 | 0 | 0 | 1 | 0 |
| 14 | 1 | 1 | 1 | 0 | 1 | 0 | 0 | 1 | 0 |
| 15 | 0 | 1 | 0 | 0 | 0 | 0 | 0 | 0.5 | 0 |
| **Results/findings** | | | | | | | | | |
| 16 | 1 | 1 | 1 | 1 | 1 | 1 | 1 | 1 | 0.5 |
| 17 | 1 | 1 | 1 | 1 | 1 | 0 | 0.5 | 1 | 0 |
| **Discussion** | | | | | | | | | |
| 18 | 1 | 1 | 1 | 1 | 1 | 1 | 1 | 1 | 0.5 |
| 19 | 1 | 1 | 1 | 1 | 1 | 0 | 1 | 1 | 1 |
| **Other** |  |  |  |  |  |  |  |  |  |
| 20 | 0 | 0 | 1 | 0 | 1 | 1 | 0 | 0 | 1 |
| 21 | 0 | 1 | 1 | 1 | 1 | 1 | 0 | 1 | 1 |
| Score* | 12 | 17.5 | 16.5 | 9.5 | 17 | 12.5 | 11 | 17 | 10.5 |

* Higher score indicates higher quality, with yes=1, partially=0.5 and no=0
