# Supplementary material for: Usability and Effectiveness of eHealth and mHealth Interventions That Support Self-Management and Health Care Transition in Adolescents and Young Adults With Chronic Disease: Systematic Review
Source: J Med Internet Res. 2024 Nov 26;26:e56556. doi: 10.2196/56556 (PMC11632288; doi:10.2196/56556)
Supplement: Multimedia Appendix 5 [file jmir_v26i1e56556_app5.docx]

| **Item** | **[34]** | **[39]** | **[40]** | **[45]** | **[46]** | **[50]** |
| --- | --- | --- | --- | --- | --- | --- |
| **Participant characteristics** | | | | | | |
| 1 | 1 | 1 | 1 | 1 | 1 | 1 |
| 2 | 0 | 0 | 0 | 0 | 1 | 1 |
| 3 | 0 | 0 | 0 | 0 | 0 | 0 |
| 4 | 1 | 1 | 1 | 1 | 0 | 1 |
| 5 | 0 | 0 | 0 | 0 | 0 | 0 |
| **Survey administration** | | | | | | |
| 6 | 0 | 1 | 0 | 1 | 1 | 1 |
| 7 | 1 | 1 | 1 | 1 | 1 | 1 |
| 8 | 0 | 0 | 0 | 1 | 0 | 0 |
| 9 | 0 | 0 | 0 | 0 | 0 | 1 |
| 10 | 0 | 0 | 0 | 0 | 0 | 0 |
| **Survey design** | | | | | | |
| 11 | 0 | 0 | 0 | 0 | 0 | 0 |
| 12 | 1 | 1 | 1 | 1 | 0 | 1 |
| 13 | 1 | 1 | 1 | 1 | 0 | 1 |
| **Data analysis** | | | | | | |
| 14 | 1 | 1 | 1 | 1 | 0 | 1 |
| 15 | N/A | N/A | N/A | N/A | N/A | N/A |
| 16 | N/A | N/A | N/A | N/A | N/A | N/A |
| Score***** | 6 | 7 | 6 | 8 | 4 | 9 |

*** Higher score indicates higher quality, with yes=1, no=0 and N/A=not applicable**
