# Supplementary material for: Usability and Effectiveness of eHealth and mHealth Interventions That Support Self-Management and Health Care Transition in Adolescents and Young Adults With Chronic Disease: Systematic Review
Source: J Med Internet Res. 2024 Nov 26;26:e56556. doi: 10.2196/56556 (PMC11632288; doi:10.2196/56556)
Supplement: Multimedia Appendix 6 [file jmir_v26i1e56556_app6.docx]

| **Study**  **identity** | **Interventin name** | **Intervention media** | **Duration** | **Intervention components** |
| --- | --- | --- | --- | --- |
| Kosse et al., 2019[31] | ADAPT | Mobile App | 6 Month | The ADAPT intervention consisted of a smartphone application for patients, which was securely connected to a desktop application of the patient's own community pharmacist, the app contained different elements targeting multiple aspects of non-adherent behavior:(1) weekly Control of Allergic Rhinitis and Asthma Test (CARAT) to monitor disease control over time; (2) short educational and motivational movies on asthma related topics; (3) medication reminder alarm; (4) peer chat function; (5) pharmacist chat function |
| Schwartz et al.,2019[32] | STEPS | Mobile App | NR | STEPS is a mobile APP with features of (1) self-management via texting, education, and plans for survivorship; (2) medication reminders; (3) upload and display documents and videos; (4) gamification via a point system to reinforce engagement; (5) texting health resources to inform, motiVate, and engage |
| Crosby et al.,2020[33] | SCThrive | Mobile App +Zoom™ | 6 Week | SCThrive consists of group sessions during which facilitators use established cognitive behavioral, motivational interviewing, and social skills strategies to target BA components. SCThrive also used unique components to enhance the developmental and cultural sensitivity of the intervention including incorporation of culturally relevant materials and newer technologies (e.g., video chat platform). All SCThrive participants were provided with a companion app, (iManage) and an iPad. Participants were trained to use iManage to record progress on their self-management goals, daily pain and mood symptoms, message other participants in between sessions, and share picture stories about their week |
| Saulsberry et al, 2020[34] | STEP | Web-Based Technology | NR | The STEP is an open-access, Web-based tool comprising 6 modules, including:(1) fundamentals of SCD disease knowledge; (2) knowledge related to disease complications; (3) necessary transition skills; (4) self-advocacy for teens with SCD; (5) Incentive (eg, small toys and board games) |
| Nichols et al, 2020[35] | SAMS | Mobile App | 2 Month | SAMS is a mobile APP with features of real-time monitoring of asthma symptoms; tracking asthma medication use; asynchronous video capture of inhaler use technique and tailored feedback provided by respiratory therapist; telehealth visits performed by an educator trained in motivational interviewing |
| Schneider et al, 2020[36] | AAP | Mobile App | 3 Month | AAP is a mobile APP with features of text reminders; peak-flow process; asthma action plan; profile options; symptom tracking and feedback on the current asthma status |
| Hood et al., 2021[37] | SCThrive | Mobile App | 6 Week | SCThrive is a multicomponent, peer-based, developmentally appropriate intervention designed to increase behavioral activation. AYA received six weekly 90-min group sessions (three in-person, three online) guided by two facilitators (psychologists, psychology fellows, or psychology graduate students. With functions of tracking symptoms; create, monitor, and complete self-management goals; culturally sensitive motivational interviewing; social skills, and cognitive behavioral strategies to enhance behavioral activation |
| Holtz et al., 2021[38] | MyT1DHero | Mobile App | 12 Week | The MyT1DHero intervention links the adolescent with T1D and their parent through 2 separate app interfaces—one for the adolescent and one for the parent. This app is designed to promote positive communication regarding T1D management. With features of a blood glucose testing schedule; communication between adolecents and parients; peer support  bonus points. In addition, there are also links to videos of other adolescents with T1D telling their stories and providing affirming messages. |
| Davis et al, 2021[39] | Kiss myAsthma | Mobile App | 6 Week | Kiss myAsthma contained eight main sections where users could interact with the app: profile; asthma history (including asthma severity, mood, asthma symptoms and exacerbations); goals; inspirations; reminders (for medication-taking or other activities); connect (i.e. to evidence-based website links); information about asthma; and emergency support (for provision of support during an asthma exacerbation). |
| Brookshire-Gay et al, 2021[40] | Roadmap 1.0 | Mobile App | NR | Roadmap 1.0 is a health information technology application, providing patients with real-time patient-specific health information from the  MiChart (Epic ; Verona, WI) electronic medical record (EMR). Modules include laboratory results (labs), medications summarized in plain language, health care provider directory, phases of HSCT care, and an interactive discharge checklist. |
| Butalia et al.,2021[41] | / | Telephone, a transition website | 18 Month | A communication technology enhanced transition coordinator intervention, which empowering the transition coordinator with the following functions:  (1)using text messaging or telephone communication to maintain contact with participants every 2 months for 12 months posttransfer;(2) Answer any questions using communication technologies; (3)Maintaining a private Facebook page and a transition website; (4) Notifying emergency visits or hospitalisations; (5) Providing supports, i.e.family physicians, financial  assistance, psychosocial supports |
| Mehta et al., 2021[42] | MedVentur | Mobile App | ~~/~~ | MedVenture is an adherence app and a survival game for both iOS and Android in which the player helps castaways survive while learning and  implementing behavioral strategies for medication taking, with specific features of autonomy support; competency support; self-identity; a parrot friend to provide company and encouragement; self-monitoring of medication adherence; game and encouraging and congratulatory messages |
| Fedele et al., 2021[43] | AIM2ACT | Mobile App | 20 Week | AIM2ACT is a dyadic mobile health intervention with several features including:(1) EMA; (2) setting asthma management goals; (3) animated skills-training videos; (4) separate videos for adolescents and caregivers  with tailored content; (5) interactive behavioral contracting; (6) medication and symptom monitoring and reminders |
| Sayegh et al.,2022[44] | Praise Text Messages | Mobile phone | 6 Week | Provided a brief text-messaging intervention, during which AYA patients  with liver transplants were praised for their efforts via text messaging  when their lab results reflected sufficient immunosuppressant medication  adherence |
| Daraiseh et al, 2022[45] | iBDecide | Mobile App | NR | Key features of iBDecide included a system for tracking medications and foods, and a novel “Treatment Generator”. This interactive feature uses a chatbot to ask questions that may influence treatment choices, such as willingness to consider injections, ability to swallow pills, and plans for college. The responses are then used to generate potential treatment options consistent with the stated preferences of the patient. Patients can read information about those options or change their answers to learn about different treatments |
| Miller et al., 2022[46] | / | A customized  website and a Mobile App | 6 Week | Including a a customized website and smartphone app. The application is a user-friendly knowledge management system which was designed to provide easier access to health-related information and resources. The application can store key personal data for the user such as allergy information, medication list, diagnosis, insurance information, and the like. In addition, it contains a reminder system for medication refills and making appointments. The application also provided resources for the user such as the ability to locate a local health care provider, employment assistance, local support groups, and postsecondary information |
| Kindem et al.,2023[47] | TusenTac®-app | Mobile App | 8 Week | The app with tailored design for SOT recipients, with features of setting the dosing time for immunosuppressants, medication reminder alerts, and the information registered in the app regarding medication intake (dose taken, the scheduled time to take the medication, the actual time of dosing), will be uploaded to a secure platform for data collection. Subsequently, the data collected on medication intake functions as self-report and allowed us to use the app as an evaluation tool of medication adherence. In addition, it can act as a medication diary. And the app was age-adapted with different fun facts appearing after a medication registration and a tailored”transplant designed” gamification system with callenges. |
| Hommel et al, 2023[48] | SMART | A portal | 8 Week | The SMART portal was comprised of 16 intervention modules, including adherence tracking, medications tracking,making and using plans, parental support, pill swallowing, overcome medication barriers, problem solving, providing reinforcement and so on. |
| Fomo et al, 2023[49] | eHARTS | Mobile App | NR | eHARTS is a mobile application used to conduct transition readiness assessments for adolescents living with HIV. Within the app users answer questions about transition readiness and receive a transition readiness score along with areas that may need improvement prior to transition |
| Chiang et al, 2022[50] | CEO | Mobile App | 4 Week | The Healthcare CEO application originally consisted of nine interfaces: (1) CEO′s Profile; (2) Health Tracking; (3) CEO Knowledge Base; (4) Barrier-free Communication; (5) See Here: Diet and Exercise; (6) Help Me, Detective!; (7) CEO Chat Room; (8) CEO′s Secretary; and (9) Who′s the Best CEO. The content of the application was customized for patients with type 1 diabetes. |
| Ghozali et al, 2023[51] | AsmaDroid® app | Mobile App | 4Week | AsmaDroid® is a mobile application with educational content and supportive functions for asthma self-management, including (1) basic asthma knowledge, (2) determining the lassification of asthma, (3) recognizing and avoiding potential triggers, (4) planning for long-term treatment, (5) appropriate asthma treatment, (6) getting regular health checkups, and (7) maintaining fitness and exercise |
| Han et al, 2023[52] | Just TRAC it | Mobile App | NR | The research nurse explained “Just TRAC it!” and emphasized this as a way to help youth manage their health care using their phones. They helped them input the following information: make a note called “My Health”; make a note for “Medications” including the name, dose, frequency, and purpose; keep a list of “Questions” to ask medical provides; keep track of “Symptoms”; record names, phone numbers, emails and addresses for health care providers; enter appointment dates and times;Take pictures of your health care card, insurance coverage card, or anything you can think of |
