# Supplementary material for: Usability and Effectiveness of eHealth and mHealth Interventions That Support Self-Management and Health Care Transition in Adolescents and Young Adults With Chronic Disease: Systematic Review
Source: J Med Internet Res. 2024 Nov 26;26:e56556. doi: 10.2196/56556 (PMC11632288; doi:10.2196/56556)
Supplement: Multimedia Appendix 7 [file jmir_v26i1e56556_app7.docx]

| **Study ID** | **Usibility tests / perceived acceptability of the intervention** |
| --- | --- |
| Schwartz et al., 2019 [32] | 1. All AYA reported that the app was easy to use and it was easy to access the messages. All but one reported that messages were useful 2. Engagement with the app ranged from 19.3% to 98.2% of app intervention days, with a median of 63.9% of days actively using the app. All AYA viewed their SCP within the app (range = 2 to 9 times; M views per participant = 5.4). All participants read text messages in the app and 3 read all of them (range of % viewed = 24-100, median = 98.4) |
| Nichols et al, 2020 [35] | The mean perceived usefulness score of the website was 7.6 whilst the hospital-based portal was  7.8. Of the 13 participants, 6 intended to use the website again whilst all intended to use the portal  again to access their medical records and e-consult |
| Schneider et al, 2020  [36] | Participants thought the app was functional and user-friendly. The majority expressed that the app assisted them with asthma selfmanagement through tracking of asthma status and text reminders to test their peak flow regularly |
| Davis et al, 2021[39] | 1. Participants reported high satisfaction with app content and usability (median score 5 out of 6 [range 4-6]) and rated the app highly on ‘feeling confident in my ability to manage my asthma’ 2. Four participants used the app one-five times, three participants used it six-ten times and two participants used it more than ten times. The data show that one user was still active well after the 6-week trial period 3. Five participants (42%) nominated goals and strategies and 3 participants (25%) entered data in the Inspiration section, a tool to support intrinsic motivation to manage asthma. |
| Brookshire-G et al, 2021[40] | 1. Among users (n = 22), engagement with Roadmap 1.0 was 96% and 91% for the first 2 weeks, respectively, and declined to 58% of those still hospitalized (n = 12) by week 4 2. Feasibility was demonstrated: 70% accessed the application. 3. Utilization was highest the first 2 weeks of hospitalization, with the laboratory results module used most |
| Fedele et al, 2021[43] | 1. Trial retention was 97% at post and 4-month visits (n ¼ 32). And retention was 100% among participants randomized to AIM2ACT 2. Both adolescents and caregivers reported being highly satisfied with AIM2ACT with content, functionality, and helpfulness, and mean satisfaction ratings for adolescents and caregivers were 4.29 and 4.25, respectively, out of a possible 5 3. On average, adolescents engaged with AIM2ACT at least once during the week for 9.59 weeks (range ¼ 3e18) during the trial period. |
| Daraiseh et al, 2022  [45] | 1. Eleven UC patients aged 14–20 completed usability testing. Participants were able to navigate through iBDecide with ease and reported that the overall look appeared “organized”, was “streamlined”, and information was “easy to access”. 2. System Usability Scale results indicated that participants on average “agree” that: ‘they would use iBDecide’ and that ‘it was easy to use and streamlined’. The mean SUS score was 78.25 (+/−12.91), range 70–90 |
| Hommel et al, 2023  [48] | 1. Usage data revealed that all participants completed at least 1 assigned intervention module (ie, all participants were not assigned all the same modules as the algorithm individually tailored   the intervention), with the highest completion rates by participants focused on IBD education (100%), medication adherence (93%), goal setting (77%), action plans (71%), and nutrition (100%)   1. Participants were assigned an average of 6 modules and the overall completion rate was 75%. |
| Fomo et al, 2023[49] | 1. Most participants found eHARTS to be acceptable because of its simplicity and lack of stigma. 2. Participants believed eHARTS was feasible as it could easily be administered within a hospital setting and integrated into regular clinic activity without disrupting patient care |
| Chiang et al, 2022 [50] | 1. The overall median score of the Questionnaire for User Interaction Satisfaction (QUIS) was 4–5, most of the 25% quartile was 4–5, and all of the 75% quartile was 5, indicating adequate user interaction satisfaction 2. over 90% of the participants indicated that the CEO application was rich in content, had multiple functions and was tailor-made for them 3. The overall median score of the QUIS was 4–5, most of the 25% quartile was 4–5, and all of the 75% quartile was 5, indicating adequate user interaction satisfaction. |
| Han et al, 2023[52] | 1. Participants felt the mobile phone apps were convenient, easy to use, and easy to access when it came to helping them manage their health. 2. The Camera, Calendar and Notes apps were the most used by participants 3. Each app was reported to be used sometimes or often by 52%-68% of participants by 6 months; at 6 months, each app was rated as very useful or extremely useful by 52%-68% of participants 4. Overall, 97% of participants stated they would recommend Just TRAC it! to others at 3 months and 100% stated they would recommend it at 6 months |
