# Supplementary material for: Usability and Effectiveness of eHealth and mHealth Interventions That Support Self-Management and Health Care Transition in Adolescents and Young Adults With Chronic Disease: Systematic Review
Source: J Med Internet Res. 2024 Nov 26;26:e56556. doi: 10.2196/56556 (PMC11632288; doi:10.2196/56556)
Supplement: Multimedia Appendix 8 [file jmir_v26i1e56556_app8.docx]

| **Interventions aim** | **Outcomes (evaluation measurement)** |
| --- | --- |
| **User engagement** |  |
| Adherence | - Usage logs - Dropout participation rate - Program adherence - Frequency of use - Time of utilization - Retention - Program compliance rate |
| **Barriers preventing usability**  Personal barriers, technical  barriers, or device barriers | - System usability test - System usability questionnaire - Technical glitches - Perceived usefulness - Missed responses - Barriers preventing engagement - Errors |
| **User feedback**  Acceptability  Satisfaction | - Enrollment and attrition rates - Evaluation questionnaire - Semistructured interview - Questionnaire for User Interaction Satisfaction (QUIS) - User interaction satisfaction - Semistructured interview |
| **Symptom control** | - Patient-Reported Symptoms - Asthma Control Test (ACT) questionnaire - Asthma Control Questionnaire |
| **Self-reported medication adherence** | - Medication Adherence Report Scale (MARS) - Assessment of Adherence to Immunosuppressive Medication Scale (BAASIS©) - 3-item visual analogue scale (VAS) - App-monitored medication adherence rate - Immunosuppressant level - Pill counts - Semistructured interview |
| **Quality of life** | - Asthma related quality of life - Pediatric Asthma Quality of Life Questionnaire (PAQLQ) - Pediatric Quality of Life Inventory |
| **Disease Knowledge** | - SCD Knowledge questionnaire - Semistructured interview |
| **Self-management development** | - Self-management confidence - Self-management skills - Self-Management efficacy |
