# Supplementary material for: Usability and Effectiveness of eHealth and mHealth Interventions That Support Self-Management and Health Care Transition in Adolescents and Young Adults With Chronic Disease: Systematic Review
Source: J Med Internet Res. 2024 Nov 26;26:e56556. doi: 10.2196/56556 (PMC11632288; doi:10.2196/56556)
Supplement: Multimedia Appendix 9 [file jmir_v26i1e56556_app9.docx]

| **Author, Year** | **Results** |
| --- | --- |
| Schwartz et al., 2019 [32] | **Usefulness**   1. The app improved health knowledge and emotional and physical well-being, had   positive impact on quality of life, related to own experiences, and provided new information not discussed with health providers; and that the applets were generally useful or interesting   1. All but one reported that messages were useful, they looked forward to receiving daily messages, and links and resources embedded in the app were helpful or interesting   **Recommendations for improvement**  Some participants suggested syncing AYA STEPS with existing health apps rather than providing applets |
| Nichols et al, 2020 [35] | **Benefits of the SAMS**   1. Facilitating autonomy among children and in transitioning from parent-managed to shared responsibility 2. Increasing competence in managing asthmatic symptoms 3. Facilitating improved medication adherence 4. Convenience 5. Facilitating more immediate communication and assessment   **Challenges of the SAMS**   1. Video upload is time-consuming 2. Bluetooth connection and proximity to the smartphone yielded challenges for families when the child did not have phone   **Recommendations for improvement**   1. Educational content 2. The ability to tailor feedback following the video capture of the use of inhaler   **Usibility**  All participating children shared the sentiment that it was easy to use and highlighted the use of technology as a normative component of their daily lives  **Needing**  Peer relationships and social acceptance |
| Schneider et al, 2020 [36] | **Recommendations for improvement**   1. Adding a larger variety of colors, backgrounds, font options, and graphics 2. Adding more customization options 3. Adding fun/entertaining elements, such as games, incentives, design elements 4. A two-way interaction option with the healthcare providers 5. Adding role model testimonials on how they cope with asthma   **App access**   1. Internet access and login information were external barriers to app use 2. Non-continuous access to a phone 3. school policy that prohibits the use of their phones while in school 4. limited free time 5. The need to carry peak-flow meter limited the opportunities for participant use of the app   **Asthma management**   1. Improving the ability and awareness to manage asthma 2. Improving medication adherence 3. Improving asthma status |
| Davis et al, 2021 [39] | 1. **Positive app attribute**   Visual appeal of the design; flexible functionality; helpful tools for goal setting, monitoring and reminders   1. **Suggestions for improvement**   Give more tips e.g. how to correctly use an inhaler; Setting goals was confusing   1. **App usefulness**   Assisted with general practitioner visits |
| Mehta et al.,2021 [42] | 1. Lack of routine contributes to nonadherence 2. Adolescents sometimes purposefully forgo medications 3. Adolescents need feeling a sense of belonging |
| Daraiseh et al.,2022 [45] | 1. The topics patients most wanted to include in a shared decision-making tool aimed at AYA patients fit into five main themes: mental health, diet/nutrition, school/work/extracurriculars, support system, and transitions. 2. The majority of patients reported using search engines (e.g., google.com) and other UC patients as their main source of information when making treatment decisions 3. Specific features patients would want included: a symptom tracker; an informal method to connect with others (e.g., AYAs with UC, healthcare providers); nutrition information and tracking; and reminders for medication and appointments |
| Miller et al.,2022 [46] | 1. All 23 adolescent participants reported that the application would be useful for managing their health information 2. Adolescents felt the smartphone application would be more helpful when complete. 3. The most desirable features of the application were that it could maintain pertinent medical information in one central location with easy access and that it contained a reminder function. 4. Drawbacks reported were that changing from one screen to the next was a challenge, and there were some functionality issues when accessing the search engines |
| Fomo et al, 2023 [49] | **Performance expectancy**   1. eHARTS was useful in determining the best time to conduct the transition readiness assessment 2. Patients with learning disabilities may have difficulty navigating the app 3. Adolescents found the app useful because it gave them the chance to complete it on his own and empower them to ask questions and know more about their health 4. eHARTS prepare them not only for the transition to adult HIV care but also for the transition from one institution to another   **Effort expectancy**   1. The app navigation features and interface were user friendly 2. The app will facilitate adolescents engaging with healthcare givers   **Facilitating conditions**   1. Problems were raised about font size, language, and the false impression eHARTS may give adolescents, which may prevent them from providing honest responses or participating in the transition assessment in general 2. Without providing feedback for adolescents to assess the level themselves may prevent them from being engaged in their own care   **Suggestion**   1. eHARTS can be framed in an empowering way as they prepare for transition to adult care 2. Adding reinforcing messages at the end of the quiz or the scores can also be presented in a way that is empowering the adolescent |
| Chiang et al, 2022 [50] | **Usefulness**  Over 90% of the participants indicated that the CEO application was rich in content, had multiple functions and was tailor-made for them.  **Suggestions**  (1) design aesthetics could be improved;  (2) the reminder music could incorporate the user′s personal database;  (3) password should be used to unlock the application to protect privacy; and  (4) concerns about a data breach if the application is logged in through Line (a predominant texting app in Taiwan). |
