# Supplementary material for: Usability and Effectiveness of eHealth and mHealth Interventions That Support Self-Management and Health Care Transition in Adolescents and Young Adults With Chronic Disease: Systematic Review
Source: J Med Internet Res. 2024 Nov 26;26:e56556. doi: 10.2196/56556 (PMC11632288; doi:10.2196/56556)
Supplement: Multimedia Appendix 10 [file jmir_v26i1e56556_app10.docx]

| **Study ID** | **Statistical evaluations of intervention efficacy** |
| --- | --- |
| Kosse et al., 2019 [39] | 1. Adherence rates of patients with low baseline adherence (MARS scores ≤19; n = 76) increased with 1.42 points in the intervention group (n = 26), adherence rates of patients in the control group (n = 50) decreased with 0.70 points. There was a positive effect of the intervention on medication adherence (MARS +2.12, *p* = 0.04). This effect was stronger (MARS +2.52, *p* = 0.02) in poor adherent adolescents with uncontrolled asthma (n = 74). 2. No effect of the intervention was observed on asthma control or quality of life |
| Crosby et al.,2020  [33] | 1. There was a clinically meaningful improvement (8-point change) in self-efficacy, with a medium effect size, *p* = 0.09, η2 = 0.06 2. With regard to HRQOL, AYA in the SCThrive intervention group had significantly higher scores than the   SCHealthEd group, *t*(32) = 2.9, *p* =0.01, *d* = 0.98   1. There was statistically significant improvement in one self-management skill (tracking health), *p* = 0.001, *d* = 0.71 2. ANOVA analyses revealed that SCD knowledge significantly increased from baseline for SCHealthEd (M = 19.52, SD = 2.99, range = 14 to 25) and SCThrive participants (M = 19.65, SD = 3.51, range = 9 to 24) compared to posttreatment for SCHealthEd (M = 20.24, SD = 3.41, range = 11 to 24) and SCThrive participants (M = 19.96, SD = 4.72, range = 7 to 25), *F*(3, 102) = 588.2, *p* < .001, η_p2_ = .95. However, there was no significant main effect of group or a group x time interaction, *p* > 0.05 |
| Saulsberry et al, 2020[34] | 1. There is a positive correlation between the number of STEP modules completed and the disease knowledge score (*P*=0.003) 2. The median disease knowledge score was significantly higher among participants who completed ≥3 STEP modules (82.7 [SD 14.68]) compared with those who completed ≤2 modules (69.57 [SD 12.82]; U=149.0; *P*=0.007) 3. There is a positive correlation (*r*=0.502) between the disease knowledge score and self-management confidence rating (*P*=0.005) |
| Hood et al.,2021  [37] | 1. Logging on to the app more frequently was associated higher mood ratings (*r* =00.54, *CI*[.18, 0.77], *p* =00.006) and lower pain ratings (*r* = −0.48, *CI*[−0.77, −0.02], *p* = 0.04) 2. Regression analyses demonstrated that after controlling for scores at baseline, the number of logins to the app predicted self-management skills (*p* = 0.05, *η^2^* = 0.17) and possibly self-efficacy (*p* = 0.08, *η^2^* = 0.13) |
| Holtz et al.,2021  [38] | 1. Diabetes care adherence significantly improved (before the study: mean 3.87 [SD 0.59]; after the study: mean 4.19 [SD 0.65]; *t_21_*=–2.52, *P*=0.02, *d*=0.52) 2. Quality of life significantly improved (before the study: mean 4.02 [SD 0.84]; after the study: mean 4.27 [SD 0.73]; t24=2.48, P=.01, d=0.32). 3. HbA1c levels (before the study: mean 8.94 [SD 1.46]; after the study: mean 8.87 [SD 1.29]; *t_24_*=0.67, *P*=0.51, *d*=0.04) and family conflict (before the study: mean 2.45 [SD 0.55]; after the study: mean 2.61 [SD 0.45]; *t_23_*=0.55, *P*=0.14, *d*=0.32) changed in the hypothesized direction, but the change was not significant 4. Higher use of the mobile app was associated with more improvement in HbA1c levels (*F_1,20_*=9.74, *P*<0.005; *R^2^* =0.33) |
| Davis et al, 2021  [39] | There was a clinically signification change in asthma quality of life (e.g. Emotional Function domain score baseline: 4.7 [2.7-6.3], follow-up: 5.7 [4.7-6.7]; *p*=0.043). |
| Butalia et al, 2021  [41] | 1. In the year following transfer, 47.1% in the usual care group vs 11.9% in the intervention group did not attend any outpatient diabetes appointments (*p* < 0.01). 2. There were no differences in glycaemic control or diabetic ketoacidosis post transfer. |
| Fedele et al.,2021  [43] | 1. Trial retention was 97% at post and 4-month visits (n ¼ 32). Notably, retention was 100% among participants randomized to AIM2ACT. 2. Asthma control significantly increased from baseline to the postintervention (*p*= 0.002) and 4-month follow-up time point (*p* < 0.001) in the AIM2ACT group, surpassing the minimally clinically important difference for this measure. There was a significant interaction effect of group and time for asthma control (*p* =0.04) 3. Asthma-related quality of life improved among adolescents in both groups. These improvements were statistically significant at the postintervention (*p* = 0.002) and 4-month follow-up time point (*p* = 0.002) among adolescents in AIM2ACT 4. Improvements in self-efficacy were statistically significant at postintervention (*p* = 0.002) and 4-month follow-up (*p* = 0.01) among adolescents in the control group |
| Sayegh et al.,2022  [44] | AYA receiving the praise text messages were significantly more likely to report taking their prescribed doses (*OR* = 2.49, *p* =0.03), taking their medicine according to the directions (*OR* = 2.39, *p* =0.04), and being highly confident in taking their medication (*OR* = 2.46, *p* = 0.04), compared to the usual services group |
| Miller et al.,2022  [46] | Knowledge of medical condition—21% increase, organization/health care system navigation—15.3% increase, identified health care provider—32% increase, and awareness of available resources—19.3% increase. |
| Kindem et al.,2023 [47] | There was no significant change in TacCV% from the year before to the year after the short-term intervention |
| Hommel et al, 2023[48] | Results from the trial demonstrated improvement in medication adherence (M = 24%–31%; *t* = 7.94, *P* < 0.05) and self-management barriers as well as trends in health-related quality of life and symptoms. |
| Ghozali et al, 2023  [51] | The results revealed a significant difference of +1.4 (*p* < 0.0001) in the pretest and posttest scores of the ACT questionnaire from the intervention group, while no difference was found in the control group |
| Han et al, 2023  [52] | 1. TRANSITION-Q scores were similar at baseline and increased over time in both groups but were not significantly different between groups 2. Each additional point at the baseline score brought, on average, a 0.7-point increase in TRANSITION-Q score (95% CI 0.5 - 0.9) at each of 3 and 6 months |
